# Complete remission of recurrent multiple insulin-producing neuroendocrine tumors of the pancreas with somatostatin analogs: a case report and literature review

**DOI:** 10.1007/s12672-022-00531-z

**Published:** 2022-07-15

**Authors:** Andreas Tartaglia, Giulia Busonero, Lorenza Gagliardi, Valentina Boddi, Federica Pieri, Maurizio Nizzoli

**Affiliations:** 1 Unit of Endocrinology and Metabolism, Morgagni Hospital, Via Carlo Forlanini 34, 47100 Forlì, Italy; 2 Unit of Pathology, Morgagni Hospital, Forlì, Italy

**Keywords:** Hypoglycemia, Multicentric insulinomas, Pancreatic neuroendocrine tumors, Somatostatin analogs

## Abstract

Hyperinsulinemic hypoglycemia is most commonly caused by a single, sporadic insulinoma. Multicentric insulinoma disease (insulinomatosis) as well as metachronous neuroendocrine tumors of the pancreas, known also as neuroendocrine adenomatosis, represent a very rare condition, if not associated with multiple endocrine neoplasia type 1 syndrome (MEN1) or Von Hippel Lindau disease. We report a 9-year follow-up of a 41-year-old woman, initially presenting with hypoglycemic syndrome caused by two insulin-producing tumors, who underwent subtotal pancreasectomy in 2012, with histology compatible with multiple small neuroendocrine tumors. An approximately 1-cm insulin-producing tumor recurred at subsequent biochemical and radiological follow-up, and was cured with the somatostatin analog octreotide as a single treatment, until remission of symptoms and complete regression of the pancreatic lesion achieved after only 16 months of treatment. The possible mechanisms for these findings are discussed and the literature is briefly reviewed.

## Introduction

Insulinomas are the most frequent cause of hyperinsulinemic hypoglycemia. They are usually benign sporadic tumors, but in about 10% of patients they may be associated with hereditary diseases, such as multiple endocrine neoplasia type 1 (MEN1) [1]. The diagnosis of insulinoma is reached by proving symptomatic hypoglycemia, generally below 50 mg/dl, in association with inappropriately elevated serum insulin (higher than 4 mU/l) and C-peptide levels during fasting, but can be established only with the criteria of the prolonged fasting test. Insulinomatosis is a very rare neoplastic condition defined by the presence of multiple small and large insulin-producing tumors, which synchronously and metachronously develop within the pancreas. In patients affected by this disease, hyperinsulinemic hypoglycemia typically recurs after removal of the visible lesions. The pathophysiology of this condition shows that the insulin-producing tumors are preceded by an insulin cell hyperplasia of the beta-cells of the pancreas [2, 3]. At present, due to the rarity of the disease, no genetic defects are known, although a genetic missense mutation has been proposed in a recent study [4].

Medical therapy for patients with neuroendocrine neoplasms (NEN), including insulin-producing ones, is aimed at decreasing the circulating hormones which cause typical syndromes and control of tumor growth.

Approximately 50% of insulinomas express somatostatin receptors on their cells’ surface, mainly receptor subtypes 2 and 5. The somatostatin analogs octreotide and lanreotide are able to inhibit the signal-transmission pathways by binding these receptors [5].

We report a case of hyperinsulinemic hypoglycemia caused by multiple recurrent small insulin-producing tumors which recurred after surgery and responded to somatostatin analog treatment with complete remission of symptoms and regression of the pancreatic lesions.

## Case report

A 41-year woman presented as an outpatient at our Hospital in November 2012 for her annual thyroid condition follow-up (simple goiter associated with autoimmune chronic thyroiditis), complaining of neurological symptoms such as poor cognitive abilities and decreased alertness over the previous two years, sometimes associated with sweating and sudden hunger. During one of these episodes, the laboratory workout revealed low capillary blood glucose levels (36 mg/dl). On the suspicion of hypoglycemic syndrome, the patient was admitted to the Endocrine Unit of Forlì Hospital. At the physical examination her weight was 70 kg and BMI 24.5; blood pressure was 110/70 mmHg and no abnormalities were found at abdominal, cardiac, pulmonary and neurological examination. Other causes of hypoglycemia were also excluded upon admission, namely exogenous insulin and oral hypoglycemic agent administration, as well as insulin autoimmune syndrome (Hirata disease), as insulin autoantibodies were negative.

**Fig. 1:**
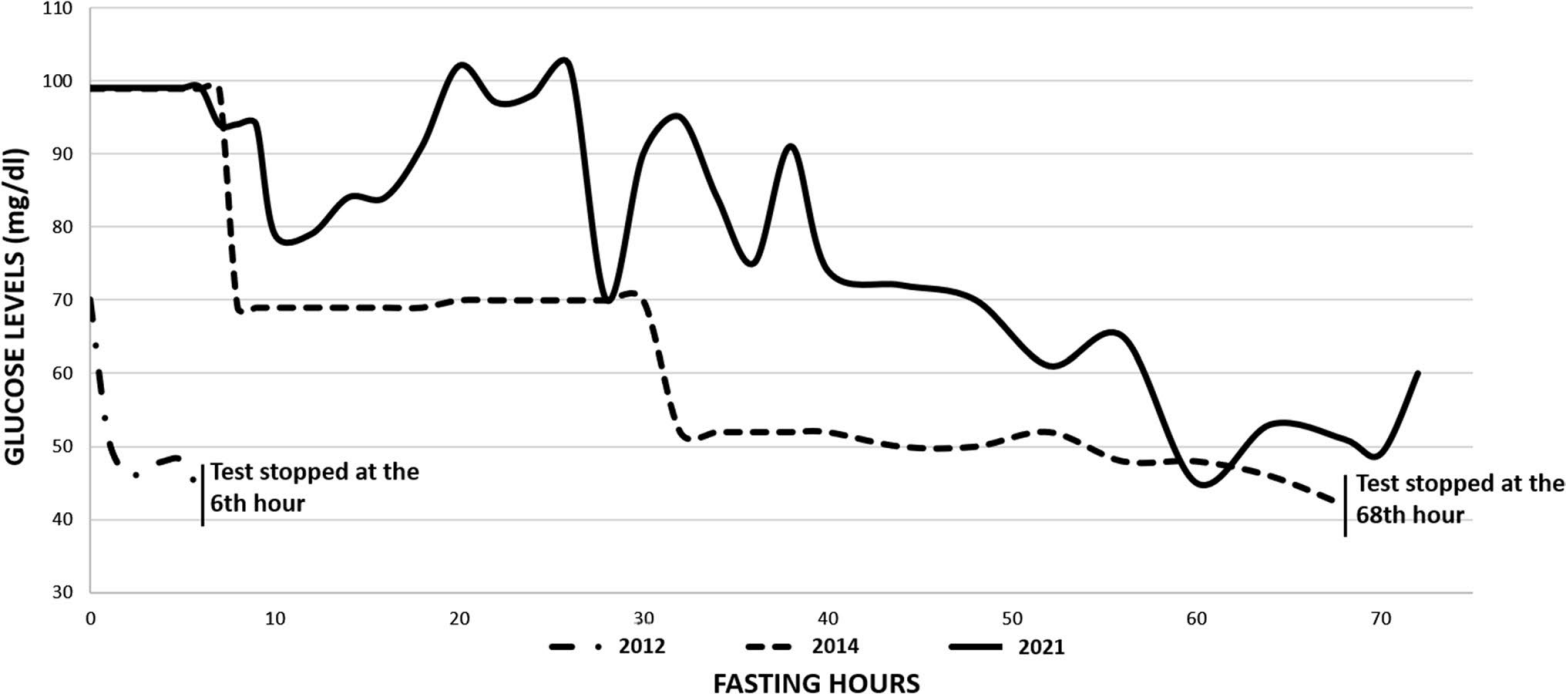
Comparison of glucose levels between 2012, 2014 and 2021 fasting tests. The latter test was not interrupted. The venous glucose values were never below 45 mg/dl and the patient showed no symptoms of hypoglycemia. (Graphic made with Microsoft Office Excel 2019 version 2205)

A supervised 72-h fasting test was performed, showing low venous glucose values (43 mg/dl; Fig. 1) and increased levels of insulin (3.7 mU/l) and C-peptide (0.43 nmol/l). The test was interrupted at the sixth hour and sugar was administered orally.

**Fig. 2:**
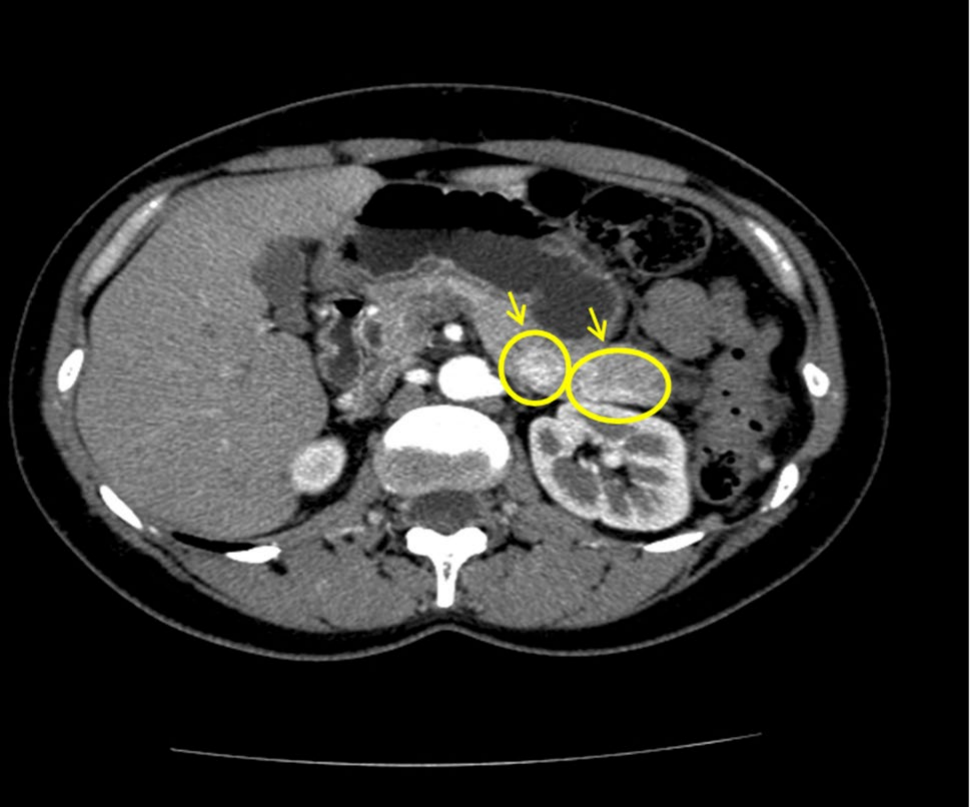
CT scan performed in 2012 showing two lesions in the body of the pancreas of 16 mm and 8 mm, respectively, with contrast enhancement features typical of insulinoma (marked with yellow arrows and circles)

An abdominal CT scan revealed 2 lesions located within the body-tail of the pancreas, measuring 16 mm and 8 mm, respectively, and presenting contrast enhancement features typical of insulinomas (Fig. 2).

**Fig. 3:**
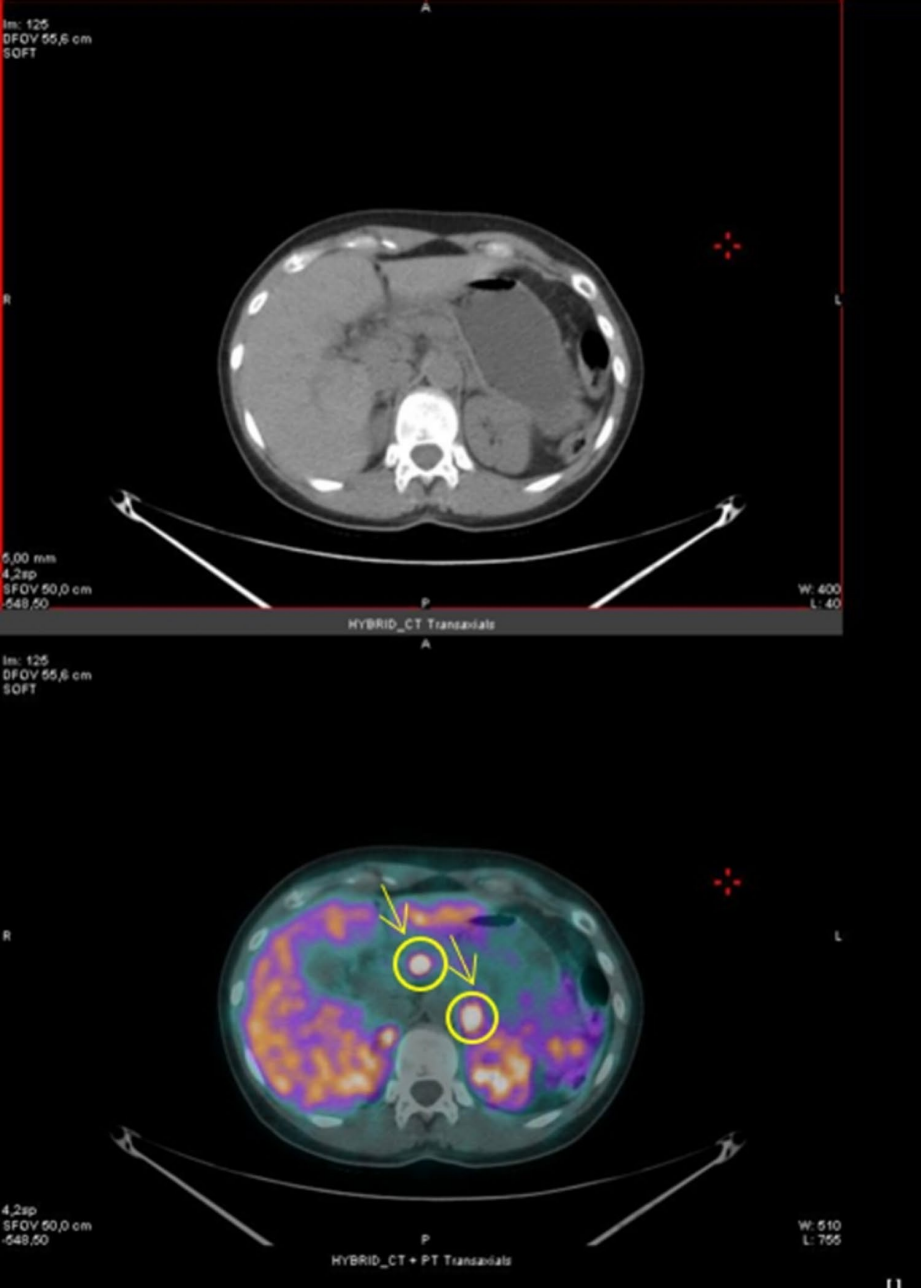
68 Ga-DOTATOC PET imaging performed in 2012, showing high expression of somatostatin receptors on the surface of the two neuroendocrine tumors of the pancreatic body with a standardized uptake value of 12 (marked in yellow arrows and circles)

Subsequently, a 68 Ga-DOTATOC positron emission tomography was performed, showing high expression of somatostatin receptors on the surface of the two above-mentioned pancreatic lesions, confirming the presence of neuroendocrine tumors (Fig. 3).

An extensive review of both personal and family history of the patient did not suggest the presence of MEN1. Circulating levels of chromogranin A (CgA), parathormone, serum calcium, insulin-like growth factor 1, as well as all pituitary hormones, i.e. prolactin, thyroid-stimulating hormone, adrenocorticotrophic hormone, luteinizing hormone, follicle-stimulating hormone and growth hormone, were normal in two different determinations. Additional diagnostic evaluations for MEN1 syndrome, such as parathyroid scintigraphy and magnetic resonance imaging (MRI) of the sella turcica, were also negative.

**Fig. 4:**
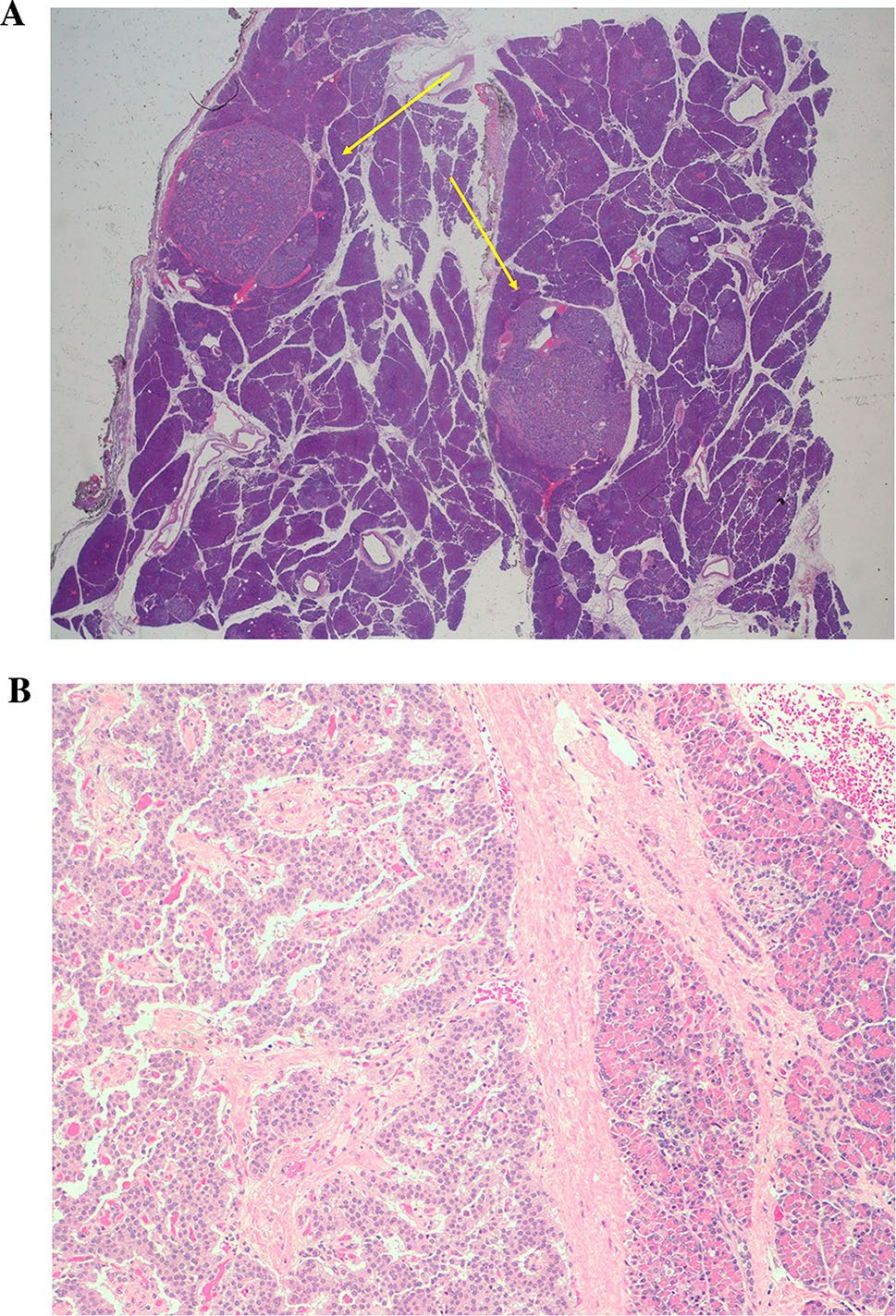
Histology of multiple neuroendocrine tumors of the pancreas with hematoxylin and eosin staining (panoramic view at 2 X power, **A**). Histology of the insulinomas at 20 X power (**B**)

**Fig. 5:**
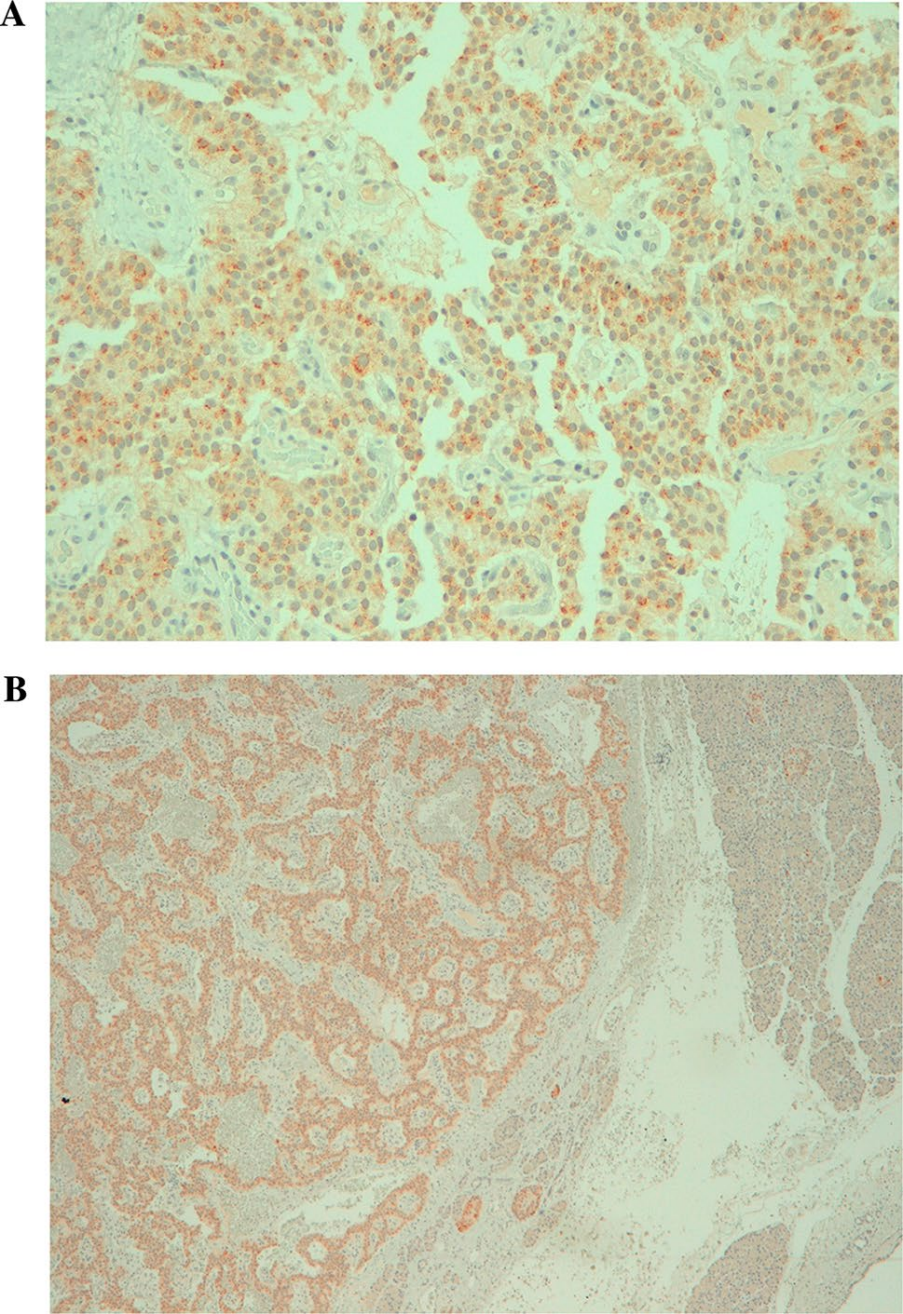
Histology of the insulinomas: immunohistochemistry staining for CgA at 20 X power (**A**) and at 5 X power (**B**). Negative control for CgA staining is displayed in endogenous cell tissue on the right side of the figure in **B**

**Fig. 6:**
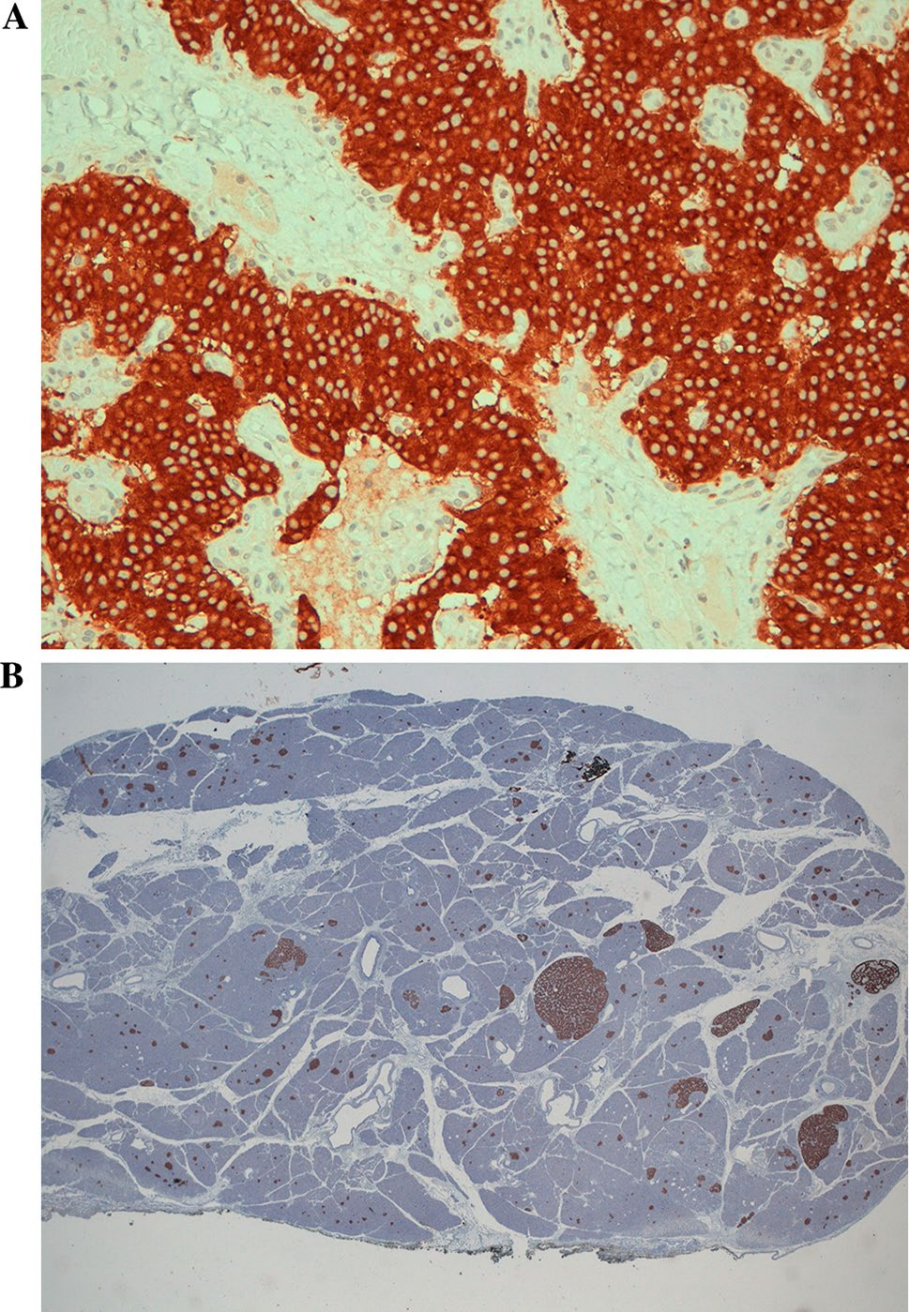
Histology of the insulinomas: immunostaining for synaptophysin staining at 20 X power (**A**) and at 2 X power. In the background, negative staining control of endogenous tissue is visible (**B**)

**Fig. 7:**
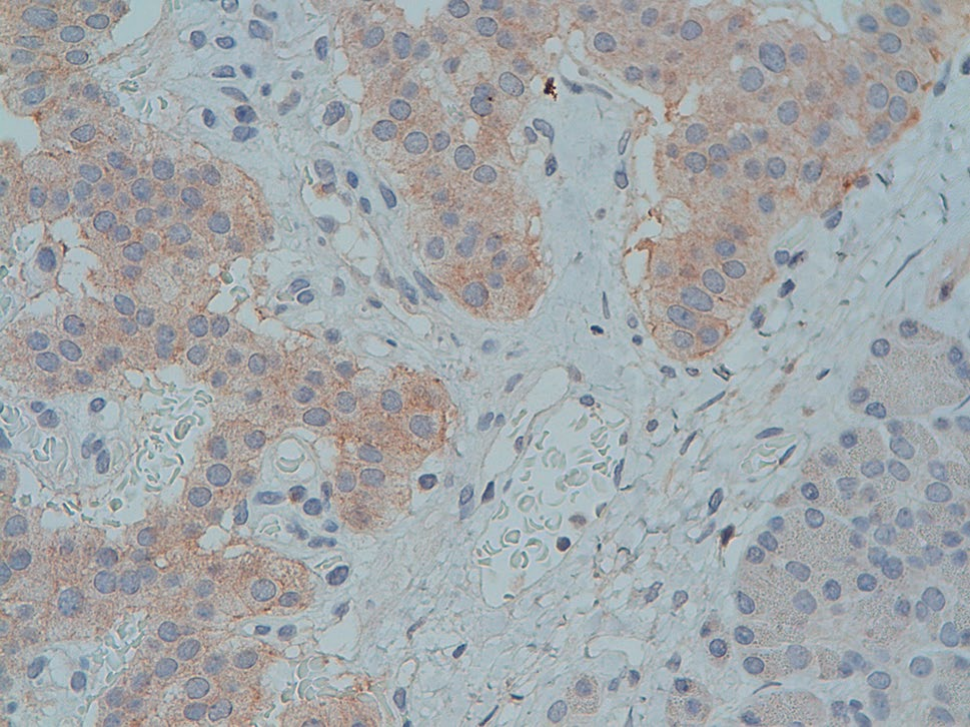
Histology of the insulinomas: immunostaining for somatostatin receptor type 2 staining at 20 X power. The expression of somatostatin receptor type 2 is mainly granular-cytoplasmatic and is incomplete on most of the tumor-cells’ surface. It is here displayed in about 95% of the neuroendocrine tumor cells. Negative control staining is visible in the pancreatic stromal cell tissue

**Fig. 8:**
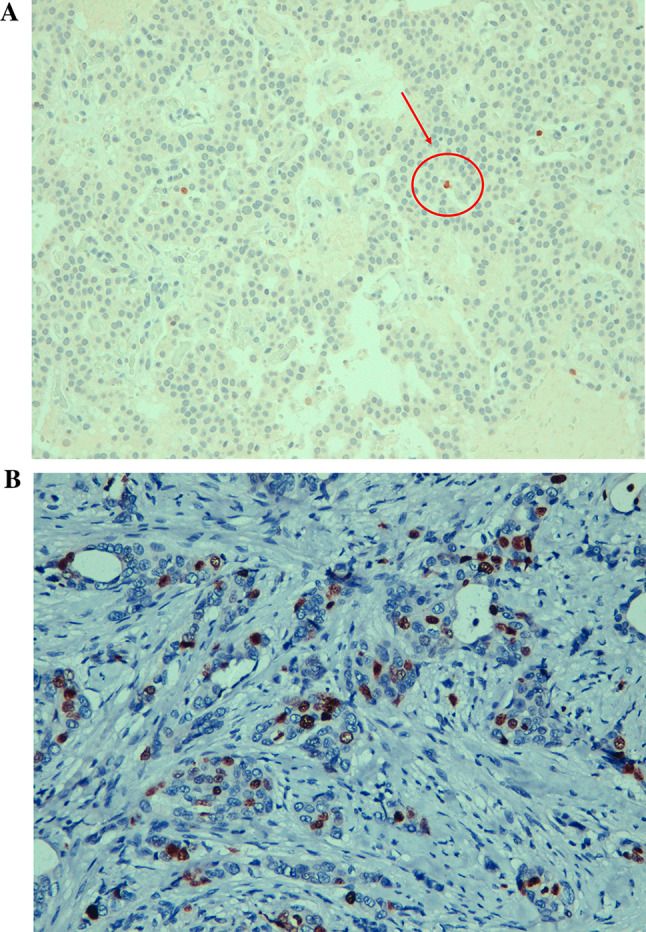
**A** Histology of the insulinomas; Ki67 immunostaining (marked with red arrow and circle): the proliferation index is 1%. **B** A positive control for ki67 staining, showing high proliferation index of about 30%

In December 2012, subtotal pancreasectomy was performed, and detailed histopathological examination revealed 2 tumors measuring 16 and 10 mm in the pancreatic body-tail along with several other small tumors (< 0.5 cm; Fig. 4A and B). The immunohistochemistry staining for CgA (clone LK2H-10, Ventana Roche Diagnostics, Fig. 5 A and B), synaptophysin (Fig. 6 A and B) and somatostatin receptors (Fig. 7) was strongly positive. Endogenous tissue background appropriate controls were provided. The proliferation activity examined with anti-Ki-67 antibody (MIB-1, DAKO, Denmark) was 1% (Fig. 8 A and B). The histology was therefore compatible with multiple well-differentiated neuroendocrine neoplasms grade 1, according to the World Health Organization (WHO) [6].

Clinically, the patient presented no symptoms of hypoglycemia, and blood sugar levels measured after surgery were within the normal range. She was therefore discharged from the surgical ward and no pharmacological treatment was given.

In January 2013, mutation analysis of the MEN1 gene was performed at the University Hospital of Ferrara, Italy, and no typical mutations of exons 2 and 10 of the MEN1 gene were observed, thus excluding the presence of MEN1 syndrome.

After almost one year (November 2013), computed tomographic scanning demonstrated a 1 cm tumor in the body of the pancreas, showing enhancing features typical of neuroendocrine lesions. Hence, a 68 Ga-DOTATOC positron emission tomography was performed, showing moderate uptake of the radioligand at the level of the above-described lesion. An FDG positron emission tomography was also performed which showed no uptake in the pancreas or other abdominal and extra-abdominal sites. Pancreatic ultrasound endoscopy confirmed the presence of a 13 mm tumor located in the body of the pancreas.

**Fig. 9:**
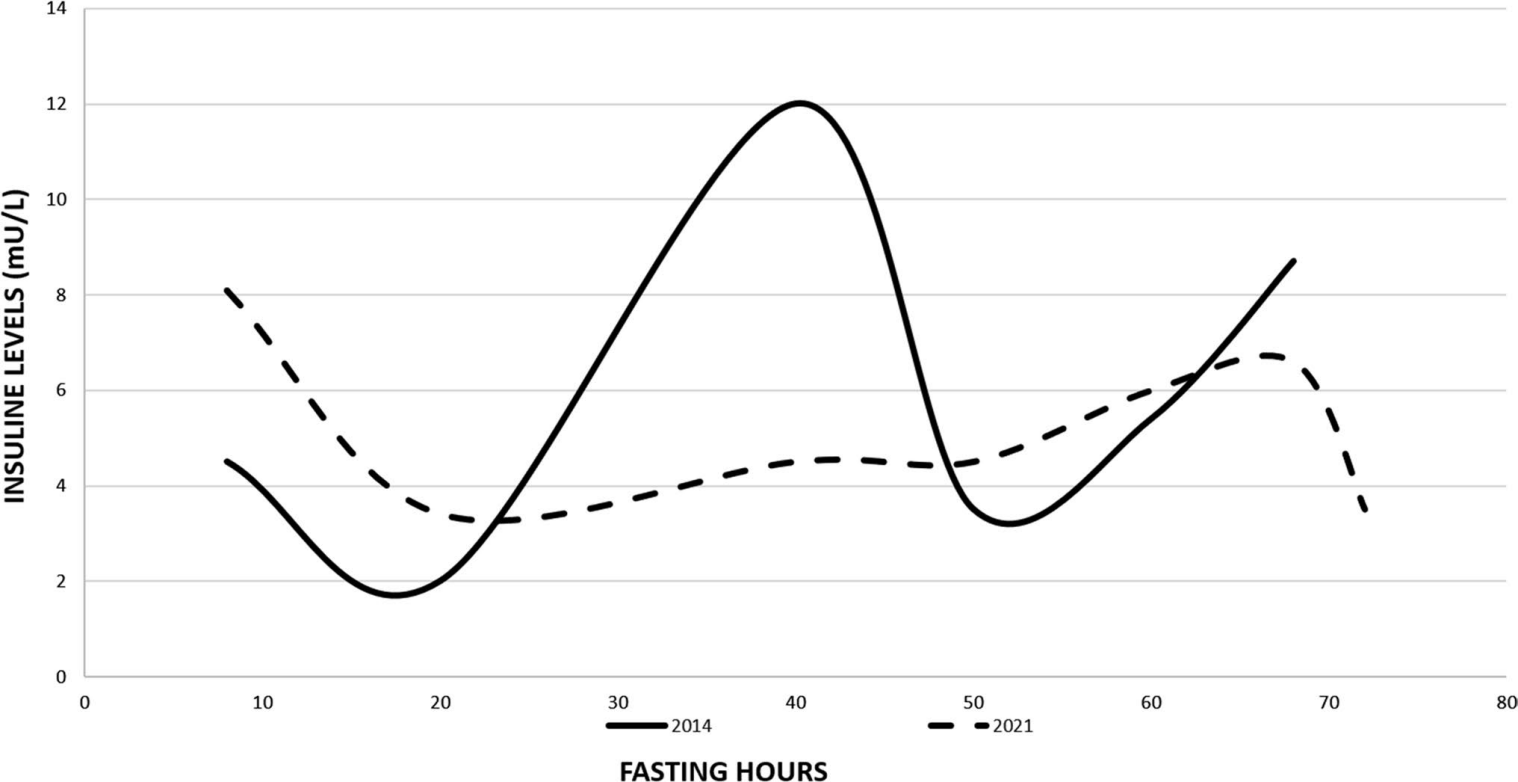
Comparison of insulin levels between 2014 and 2021 fasting tests. In the latter test plasma levels of insulin were remarkably lower than in the first fasting test. (Graphic made with Microsoft Office Excel 2019 version 2205)

In January 2014, another supervised 72-h fasting test was performed at the Endocrine Unit of Forlì Hospital, showing low venous glucose values (42 mg/dl; Fig. 1) and increased levels of insulin (8.7 mU/l; Fig. 9) and C-peptide (0.43 nmol/l). The test was interrupted at the sixty-eighth hour (Fig. 1), even though the patient presented no symptoms of hypoglycemia.

The case was repeatedly discussed by the Multidisciplinary Medical Team for Management and Treatment of Neuroendocrine Tumors of Romagna both in January and February 2014. Various medical strategies were suggested, including a second surgical approach, radiolabeled target therapy and diazoxide treatment. However, the patient refused to undergo further surgery and other specific treatments with possible side effects. In February 2014, after seeking internationally recognized professional expert opinion (Kjell Öberg, University Hospital of Uppsala, Sweden), medical treatment with the somatostatin analog octreotide (30 mg given intramuscularly every 28 days) was started and regularly administered during the subsequent follow-up.

In the following 6 months, the patient did not show any symptoms or signs of hypoglycemia and the treatment with octreotide was tolerated without particular side effects. The plasma levels of CgA, glycemia and insulin were within the normal range. The abdominal MRI performed in July 2014 showed a 12 mm angioma of the liver and apparently no lesions of the pancreas.

In November 2015, a CT scan of the abdomen was performed, showing no lesions within the pancreas and confirming the presence of a stable angioma of the liver.

A second opinion was given by the Oncological Centre of Milan, confirming the treatment with somatostatin analogs, which was eventually discontinued in September 2016, because of the patient’s desire for pregnancy.

A 68 Ga-DOTATOC positron emission tomography performed in September 2018 showed no uptake of the radioligand.

In October 2018, pancreatic ultrasound endoscopy confirmed the absence of lesions in the head-body of the pancreas.

**Fig. 10:**
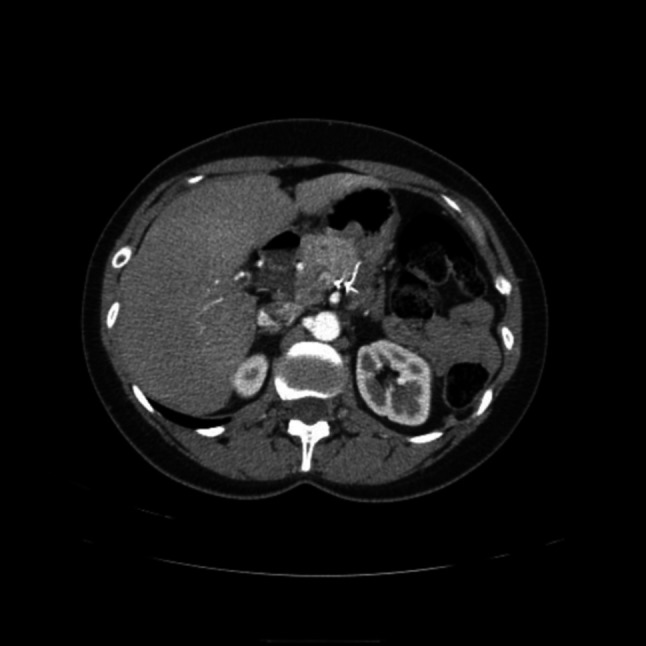
CT scan performed in April 2021, i.e., 6 years and 8 months after stopping somatostatin analog treatment, showing no lesions of the pancreas

In the following years until April 2021, the patient continued with regular 6-month clinical check-ups, including measurements of basal glycemia, insulin, C-peptide and CgA, which were always unremarkable, and annual imaging techniques, both with CT scans of the abdomen (Fig. 10) and MRI of the abdomen. All the results were completely negative for recurrent disease.

In April 2021 the patient was admitted to Endocrine Unit of Forli Hospital in order to once again perform a supervised 72-h fasting test, about 9 years after the first one: the venous glucose values were never below 45 mg/dl and the levels of insulin (4.5 mU/l) and C-peptide (0.26) measured at the lowest sugar levels were not consistent with recurrency (Figs. 1 and 9). The test was not interrupted and the patient showed no symptoms of hypoglycemia, indicating complete remission of the insulinoma syndrome.

The patient now continues with the regular follow-up, remaining asymptomatic and taking no specific medical treatment.

## Discussion

To our knowledge, this is the first described case of a primary neuroendocrine tumor of the pancreas without metastasis that was completely cured with medical therapy after recurrent disease. We report a rare case of severe hypoglycemia caused by multiple small insulin-producing neoplasms which recurred after surgery and responded to somatostatin analog treatment with complete remission of symptoms and regression of recurrent disease.

Insulinomas, which are the most frequent cause of the hypoglycemic syndrome due to hyperinsulinemia, are generally benign tumors. The median age at onset of the disease is 47 years and the survival rate is 100% at the 5-years follow-up, although malignant behavior is sometimes displayed in a few cases. In about 10% of patients, insulinoma can be associated with MEN1 and very infrequenty with Von Hippel-Lindau syndrome. It must be suspected when patients present with symptoms or signs consistent with hypoglycemia, documented by plasma glucose levels less than 50 mg/dl, and relief of symptoms after eating or intravenous glucose administration (the so-called Whipple’s triad). Hypoglycemic episodes occur mostly during fasting periods and sometimes during the post-prandial phase [7, 8]. In order to confirm the diagnosis, a supervised 72-h fasting test is required [9], although a 48-h fasting test has also been suggested [10].

Another interesting and very rare pathological condition in our case is the presence of pancreatic neuroendocrine microadenomatosis and, more specifically, insulinomatosis. This is a neoplastic pancreatic disease defined by the presence of multiple small and large insulin-producing tumors which develop simultaneously or metachronously [11]. The recurrency of hyperinsulinemic hypoglycemia after the surgical removal of all the detectable pancreatic lesions is very typical in this rare condition [12]. The tumors likely develop from an insulin-cell hyperplasia of the beta-cells of the pancreas which is responsible for the hypoglycemic syndrome.

Pancreatic neuroendocrine lesions equal to or less than 0.5 cm are defined neuroendocrine microadenomas. Multiple neuroendocrine microadenomas are called neuroendocrine adenomatosis. The prevalence of this condition is very low and may occur sporadically or associated with the above-mentioned genetic syndromes as for insulinomatosis [13]. As for all the neuroendocrine tumors, even microadenomatosis may be clinically silent or present with typical hypersecretive syndrome, as described by Alencar et al. [14].

Somatostatin receptors are expressed on the cell surface of approximately 80% of NENs and 50% of insulinomas, allowing somatostatin analogs octreotide and lanreotide to bind to receptor subtypes 2 and 5 [15]. Thanks to the inhibition of the signal-transmission pathways mediated by somatostatin receptors, tumor-related syndromes are ameliorated and tumor growth is stabilized [16].

Somatostatin analogs are the second-line medical treatment for controlling hypoglycemia in patients with insulinomas, mainly for malignant insulinomas with recurrent hypoglycemic events. Octreotide was shown to be effective in controlling hypoglycemia in 59% of patients with insulinomas [17]. Other medical treatment options include the other first-generation somatostatin analog lanreotide LAR, the second-generation somatostatin analog pasireotide, as well as everolimus and chemotherapy, including temozolamide. However, in insulinomas without somatostatin receptor expression, somatostatin analogs may worsen hypoglycemia by inhibiting counter-regulatory mechanisms, namely glucagon and growth hormone release [5].

Although somatostatin analogs were developed primarily for reducing the levels of circulating hormones causing typical endocrine syndromes, the results of PROMID [18] and CLARINET [19] open-label studies confirmed that somatostatin analogs have a positive impact on progression-free survival in pancreatic and non-pancreatic neuroendocrine tumors, due to their anti-proliferative effect [20]. This is due to somatostatin receptor density on the neoplastic cells that allow the treatment with somatostatin analog octreotide to be effective, as shown in our patient.

Interestingly, in our case not only was there a complete remission of the symptoms after starting the somatostatin analog octreotide, but the antiproliferative effect on the 10 mm lesion of the pancreas was also observed. As a matter of fact, no lesions were visible at abdominal CT scan or MRI, nor at endoscopic ultrasonography, which has a very high diagnostic accuracy of about 98%, after a total of 16 months of treatment.

The possible mechanisms of this unexpected achievement with biotherapy in our patient have to be sought in the quite exceptional overexpression of somatostatin receptor type 2, with a density of about 95% on the neoplastic surface cells, as demonstrated by immunohistochemistry examination (Fig. 7) and at 68 Ga-DOTATOC PET imaging, which is not frequent in insulin-producing tumors. Additionally, octreotide generally shows high affinity for the somatostatin receptor type 2. The very low proliferative index ki67 must have played an important role. The antisecretory effects of octreotide occur through the inhibition of specific enzymes (adenylyl cyclase) and specific voltage-dependent calcium channels, whereas the direct antiproliferative effects are related to the activation of specific phosphatases which trigger intracellular pro-apoptotic signals and induce the phosphorylation of protein tyrosine phosphatase receptors, impairing cell proliferation [16]. Somatostatin analogs also display indirect antiproliferative effects through the inhibition of circulating growth factors such as vascular endothelial growth factor, insulin-like growth factor 1 and 2, as well as through the inhibition of tumor angiogenesis [16].

We therefore believe that further studies should investigate the role of biotherapy with somatostatin analogs in newly-diagnosed primary well-differentiated neuroendocrine tumors for clinical practice.

## Summary

In summary, we describe a rare case of a patient with hypoglycemic syndrome caused by multiple neuroendocrine tumors of the pancreas (insulinomatosis). The response obtained by antineoplastic treatment resulted in tumor load decrease up to complete remission of the disease; the use of somatostatin analogs in treating single or multifocal insulinomas as well as functioning and non-functioning neuroendocrine multiple subcentimetric tumors should thus be considered as a pharmaceutical option, when surgery is not possible or surgical eradication is not achieved.

## Data Availability

The datasets generated during and/or analyzed during the current study are available from the corresponding author on reasonable request.

## References

[1] Falconi M, Eriksson B, Kaltsas G, Bartsch DK, Capdevila J, Caplin M, Kos-Kudla B, Kwekkeboom D, Rindi G, Klöppel G, Reed N, Kianmanesh R, Jensen RT (2016). ENETS consensus guidelines update for the management of patients with funcional pancreatic tumors and non-functional pancreatic tumors. Neuroendocrinology.

[2] Anlauf M, Bauersfeld J, Raffel A, Koch CA, Henopp T, Alkatout I, Schmitt A, Weber A, Kruse ML, Braunstein S, Kaserer K, Brauckhoff M, Dralle H, Moch H, Heitz PU, Komminoth P, Knoefel WT, Perren A, Klöppel G (2009). Insulinomatosis: a multicentric insulinoma disease that frequently causes early recurrent hyperinsulinemic hypoglycemia. Am J Surg Pathol.

[3] Klöppel G, Anlauf M, Perren A, Sipos B (2014). Hyperplasia to Neoplasia sequence of duodenal and pancreatic neuroendocrine diseases and pseudohyperplasia of PP-cells in the pancreas. Endocr Pathol.

[4] Iacovazzo D, Flanagan SE, Walker E, Quezado R, de Sousa Barros FA, Caswell R, Johnson MB, Wakeling M, Brändle M, Guo M, Dang MN, Gabrovska P, Niederle B, Christ E, Jenni S, Sipos B, Nieser M, Frilling A, Dhatariya K, Chanson P, de Herder WW, Konukiewitz B, Klöppel G, Stein R, Korbonits M, Ellard S (2018). MAFA missense mutation causes familial insulinomatosis and diabetes mellitus. Proc Natl Acad Sci USA.

[5] Maton PN (1993). Use of octreotide acetate for control of symptoms in patients with islet cell tumors. World J Surg.

[6] Rindi G, Klimstra DS, Abedi-Ardekani B, Asa SL, Bosman FT, Brambilla E, Busam KJ, De Krijger RR, Dietel M, El-Naggar AK, Fernandez-Cuesta L, Klöppel G, McCluggage WG, Moch H, Ohgaki H, Rakha EA, Reed NS, Rous BA, Sasano H, Scarpa A, Scoazec JY, Travis WD, Tallini G, Trouillas J, Van Krieken JH, Cree IA (2018). A common classification frame work for neuroendocrine neoplasms: an International Agency for Research on Cancer (IARC) and World Health Organization (WHO) expert consensus proposal. Mod Pathol.

[7] Cryer PE, Axelrod L, Grossman AS, Heller SR, Montori VM, Seaquist ER, Service FJ (2009). Evaluation and management of adult hypoglycemic disorders: an endocrine society clinical practice guidelines. J Clin Endocrinol Metab.

[8] Vezzosi D, Bennet A, Fauvel J, Caron P (2007). Insulin, C-peptide and proinsulin for the biochemical diagnosis of hypoglycaemia related to endogenous hyperinsulinism. Eur J Endocrinol.

[9] Placzkowski KA, Vella A, Thompson GB, Grant CS, Reading CC, Charboneau JW, Andrews JC, Lloyd RV, Service FJ (2009). Secular trends in the presentation and management of functioning insulinoma at the Mayo Clinic, 1987–2007. J Clin Endocrinol Metab.

[10] Hirschberg B, Livi A, Bartlett DL, Libutti SK, Alexander HR, Doppman JL, Skarulis MC, Gorden P (2000). Forty-eight-hour fast: the diagnostic test for insulinoma. J Clin Endocrinol Metab.

[11] De Sousa SM, Haghighi KS, Qiu MR, Greenfield JR, Chen DLT (2016). Synchronous nesidioblastosis, endocrine microadenoma, and intraductal papillary mucinous neoplasia in a man presenting with hyperinsulinemic hypoglycemia. Pancreas.

[12] Vozxv N, David W, Cohen DW, Dillhoff ME, Jin M (2017). Pancreatic neuroendocrine microadenomatosis: a case report of cytology and histology correlation. Diagn Cytopathol.

[13] Babic B, Keutgen X, Nockel P, Miettinen M, Millo C, Herscovitch P, Patel D, Nilubol N, Cochran C, Gorden P, Kebebew E (2016). Insulinoma due to multiple pancreatic microadenoma localized by multimodal imaging. J Clin Endocrinol Metab.

[14] Alencar N, Nunes M, Segatelli V, Castanheira T, Seraphim C, Pereira M (2019). SUN-320 insulinomatosis: a rare cause of recurrent hyperinsulinemic hypoglycemia. J Endocr Soc.

[15] Vezzosi D, Bennet A, Rochaix P, Courbon F, Selves J, Pradere B, Buscail L, Susini C, Caron P (2005). Octreotide in insulinoma patients: Efficacy on hypoglycemia, relationship with Octreoscan scintigraphy and immunostaining with anti-sst2A and anti-sst5 antibodies. Eur J Endocrinol.

[16] Gomes-Porras M, Càrdenas-Salas J, Alvarez-Escolà C (2020). Somatostatin analogs in clinical practice: a review. Int J Mol Sci.

[17] Grimaldi F, Fazio N, Attanasio R, Frasolari A, Papini E, Cremonini N, Davì MV, Funicelli L, Massironi S, Spada F, Toscano V, Versari A, Zini M, Falconi M, Öberg K (2018). Assessment of response to treatment and follow-up in gastroentero-pancreatic neuroendocrine neoplasms. Endocr Metab Immune Disord Drug Targets.

[18] Rinke A, Muller HH, Schade-Brittinger C, Klose KJ, Barth P, Wied M, Mayer C, Aminossadati B, Pape UF, Bläker M, Harder J, Arnold C, Gress T, Arnold R (2009). Placebo-controlled, double-blind, prospective, randomized study on effect of octreotide LAR in the control of tumor growth in patients with metastatic neuroendocrine midgut tumors: a report from the PROMID study group. J Clin Oncol.

[19] Caplin ME, Pavel M, Cwila JB, Phan AT, Raderer M, Sedláčková E, Cadiot G, Wolin EM, Capdevila J, Wall L, Rindi G, Langley A, Martinez S, Blumberg J, Ruszniewski P (2016). Anti-tumour effect of lanreotide for pancreatic and intestinal neuroendocrine tumours: the CLARINET open-label extension study. Endocr Relat Cancer.

[20] Tomassetti P, Migliori M, Caletti GC, Fusaroli P, Corinaldesi R, Gullo L (2000). Treatment of type II gastric carcinoid tumors with somatostatin analogues. N Engl J Med.

